# Response of the multiple-demand network during simple stimulus discriminations

**DOI:** 10.1016/j.neuroimage.2018.05.019

**Published:** 2018-08-15

**Authors:** Tanya Wen, Daniel J. Mitchell, John Duncan

**Affiliations:** Medical Research Council, Cognition and Brain Sciences Unit, 15 Chaucer Road, Cambridge CB2 7EF, United Kingdom

**Keywords:** Multiple-demand network, Difficulty, Motion coherence, Sensory, Attention

## Abstract

The multiple-demand (MD) network is sensitive to many aspects of task difficulty, including such factors as rule complexity, memory load, attentional switching and inhibition. Many accounts link MD activity to top-down task control, raising the question of response when performance is limited by the quality of sensory input, and indeed, some prior results suggest little effect of sensory manipulations. Here we examined judgments of motion direction, manipulating difficulty by either motion coherence or salience of irrelevant dots. We manipulated each difficulty type across six levels, from very easy to very hard, and additionally manipulated whether difficulty level was blocked, and thus known in advance, or randomized. Despite the very large manipulations employed, difficulty had little effect on MD activity, especially for the coherence manipulation. Contrasting with these small or absent effects, we observed the usual increase of MD activity with increased rule complexity. We suggest that, for simple sensory discriminations, it may be impossible to compensate for reduced stimulus information by increased top-down control.

## Introduction

Diverse studies examining a range of cognitive demands have found of a set of frontal-parietal regions that are consistently involved in a variety of tasks, ranging from response inhibition to working memory to decision making (e.g., Duncan and Owen, 2000; Fedorenko et al., 2013; Niendam et al., 2012; Stiers et al., 2010). Included in this pattern are regions of the dorsolateral prefrontal cortex, extending along the inferior/middle frontal gyrus (IFG/MFG), and including a posterior-dorsal region close to the frontal eye field (pdLFC), parts of the anterior insular cortex (AI), pre-supplementary motor area and adjacent anterior cingulate cortex (pre-SMA/ACC), and intraparietal sulcus (IPS). Together they have been termed the multiple demand (MD) network (Duncan, 2010), cognitive control network (Niendam et al., 2012), or task positive network (Fox et al., 2005).

Activity in the MD network increases with increases in many kinds of task difficulty or demand, such as with additional subgoals (e.g., Farooqui et al., 2012), greater working memory demand (Manoachet al., 1997), resisting strong competitors (e.g., Baldauf and Desimone, 2014), task switching (e.g., Wager et al., 2004), or a wide range of other task demands (e.g., Crittenden and Duncan, 2014; Jovicich et al., 2001; Marois et al., 2004; Woolgar et al., 2015a). Increased activity in more difficult conditions can also be accompanied by stronger information coding, shown by multivoxel pattern analysis (e.g., Woolgar et al., 2015a; Woolgar et al., 2011; Woolgar et al., 2015b). Reflecting these widespread effects of demand, the MD network has been suggested to implement top-down attentional control, optimally focusing processing for the requirements of a current task (Miller and Cohen, 2001; Duncan, 2010; see also Norman and Shallice, 1980).

One simple way to manipulate task difficulty is through the quality of stimulus information. Some experiments have shown clear MD responses as stimulus discriminability decreases (e.g., Crittenden and Duncan 2014; Deary et al., 2004; Holcomb et al., 1998; Jiang and Kanwisher, 2003; Sunaert et al., 2000; Woolgar et al., 2011), but this has not always been the case (Cusack et al., 2010; Dubis et al., 2016; Han and Marois, 2013; Muller-Gass and Schroger, 2007). For example, Cusack et al. (2010) contrasted hard and easy trials of a task in which participants had to detect a barely perceptible ripple in an oscillating dot field and found no neural activation differences between the two sensory difficulty levels, despite substantial differences in behavioral performance, and robust BOLD contrast to a different task manipulation (attention switching).

In an important study, Han and Marois (2013) investigated activity in parts of the MD system during a task in which three letter targets were to be identified in a rapid stream of digit nontargets. In the baseline condition, the three letters occurred in immediate succession; to increase demand, they either inserted a nontarget into the series of three targets, or reduced exposure duration. While activity in frontal-parietal areas increased with the addition of a distractor, exposure duration had little effect. To interpret their findings, Han and Marois (2013) appealed to the distinction made by Norman and Bobrow (1975), between data-limited and resource-limited behavior. Norman and Bobrow (1975) proposed that, for any task, some function (the performance-resource function or PRF) relates performance to investment of attentional resources. When this function is increasing, behavior is said to be resource-limited, and additional investment is repaid by improved performance. When the function asymptotes, further investment has no positive effect, and performance is said to be data-limited. In line with a link of MD activity to attentional investment, Han and Marois (2013) used these ideas of data- and resource-limitation to explain their findings. They proposed that, in their task, brief exposure duration created data limits, which could not be offset by increased fronto-parietal recruitment, while adding a distractor introduced resource limits by calling for increased attentional focus.

**Fig. 1:**
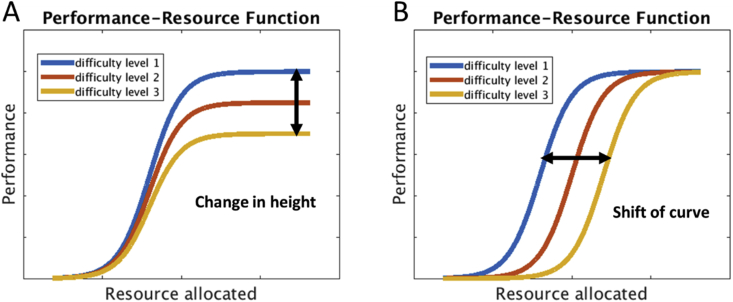
Theoretical performance-resource function (PRF) plots. (A). Difficulty might change the asymptote of the PRF. Increased difficulty cannot be offset by increased resource allocation. (B). Difficulty might shift the PRF. Increased difficulty can be offset by increased resource allocation.

In general it is not known when performance will be resource- or data-limited, but within this general framework, many patterns of results are possible. Fig. 1A illustrates a case in which, as difficulty level varies, there is no reason to expect increased attentional allocation. In this case, PRFs asymptote at different performance levels for the different levels of task difficulty, but across difficulty levels, the asymptote occurs at the same level of allocated resource. Fig. 1B illustrates an opposite case, with increased task difficulty potentially offset by increased resource allocation. This uncertainty over the role of attentional investment in different cases of perceptual discrimination could help to explain disparate results in the literature, with some cases (e.g. Han and Marois, 2013, manipulation of exposure duration) more resembling Fig. 1A, and others (e.g. Han and Marois, 2013, distractor manipulation) more resembling Fig. 1B.

In our first experiment, we sought to strengthen the evidence that, for simple sensory discriminations, MD activity can be rather independent of task difficulty, providing an exception to the “multiple demand” pattern. For this purpose we used a motion discrimination task with two kinds of difficulty manipulation – motion coherence and salience of task-irrelevant dots. For the strongest possible effect, we manipulated both variables over a wide range, moving performance from close to ceiling to close to chance. In the task demand literature, several studies have shown that, as opposed to a monotonic increase of MD activity with task difficulty, there was an inverted U-shape response (Callicott et al., 1999; Linden et al., 2003), or a plateau after a certain difficulty level (Marois and Ivanoff, 2005; Todd and Marois, 2004; Mitchell and Cusack, 2008). A possible interpretation is that MD activity initially increases with task demands, but plateaus or even declines once the task becomes impossible even with maximal attention. In our study we examined MD activity over the full range of possible task difficulties.

In addition to manipulating both aspects of difficulty over a wide range, between participants we varied whether difficulty levels were mixed or blocked. In the mixed design, levels of difficulty were presented in random order, without advance cueing of the level to be experienced on a given trial. In contrast, difficulty level was known in advance in the blocked design. With this manipulation, we asked whether MD activity is driven more proactively, by expectancy of forthcoming demand, or more reactively, when high demand is experienced on a current trial.

Finally, in modeling our fMRI data, we attempted to remove effects of decision time, expected to increase with either sensory or selection difficulty. In two prior studies of motion coherence, trials were modelled simply as events, without regard for their duration (Kayser et al., 2010a, 2010b). In this case, greater brain activity associated with decreasing motion coherence may simply have reflected longer processing times. To diminish such effects, our fMRI model explicitly included decision time for each trial.

Though PRF shapes are generally unknown, our use of two different demand manipulations afforded the possibility of different outcomes. In particular, we expected that top-down control could be especially important in the irrelevant-dots condition, leading to larger effects of demand on MD activity. Though Experiment 1 showed results in line with this expectation, they occurred against a background of generally weak effects, and no significant difference between the two manipulations. In Experiment 2 we reexamined coherence and irrelevant-dots conditions in a new group of participants, and compared these sensory demands with a manipulation of rule complexity.

## Methods

### Experiment 1

#### Participants

Participants were randomly assigned to either the blocked or mixed group, with this variable manipulated between participants to minimize carryover effects. A total of 40 participants took part in the experiment. Twenty-one participants (9 male, 12 female, ages 19–31, mean = 25.7) took part in the blocked group, and nineteen participants (11 male, 9 female, ages 19–36, mean = 23.9) took part in the mixed group. Participants were recruited from the volunteer panel of the MRC Cognition and Brain Sciences Unit and paid to take part. An additional 16 participants were excluded (10 participants had excessive motion > 5 mm, and another 6 had poor performance with accuracies more than three median absolute deviations below the median in at least one condition). All participants were neurologically healthy, right-handed, with normal hearing and normal or corrected-to-normal vision. Procedures were carried out in accordance with ethical approval obtained from the Cambridge Psychology Research Ethics Committee, and participants provided written, informed consent before the start of the experiment.

#### Experimental setting and design

Each participant performed two conditions of a motion coherence task, referred to here as the coherence condition and the irrelevant-dots condition. Each condition spanned six levels of difficulty. Difficulty type and level served as within-subject factors. This resulted in a group (blocked vs. mixed) × difficulty type (coherence vs. irrelevant-dots) × difficulty level (level 1 ∼ level 6) design.

#### Stimuli and procedures

**Fig. 2:**
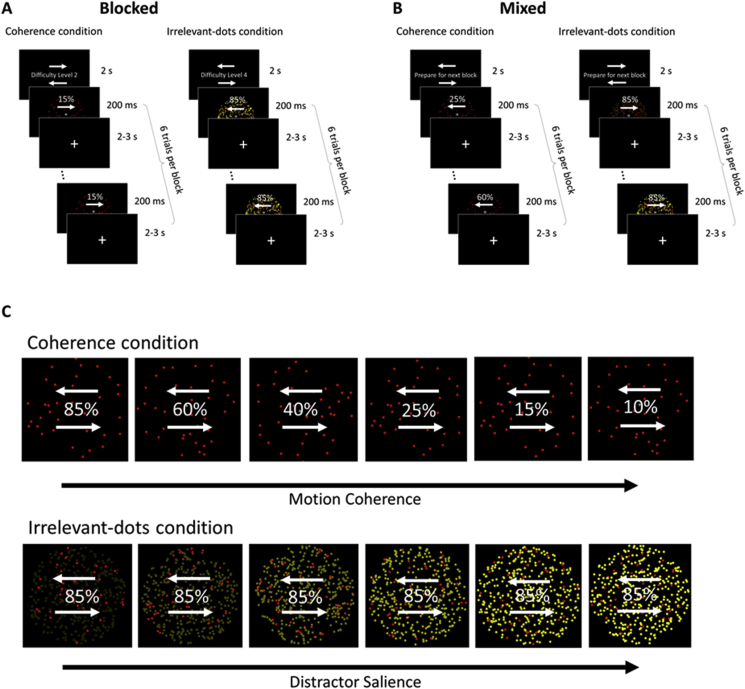
An example of one block of each condition in the blocked design (A) and the mixed design (B). At the beginning of each block, participants were given the response mappings and were cued to prepare for that block. At the beginning of each trial, a 200 ms RDK appeared, followed by a 2–3 s fixation period, during which participants were to judge the motion direction of the red dot field. The percentages indicate degree of motion coherence and were not shown on the actual display. (C) An illustration of the 6 levels of the coherence and irrelevant-dots conditions. In the coherence condition, difficulty was manipulated by decreasing the motion coherence of the dot field. In the irrelevant-dots condition, difficulty was manipulated by increasing the salience of the yellow dots. The percentages indicate the degree of motion coherence and were not shown on the actual display.

The task structure was similar for both blocked and mixed designs (see Fig. 2). On each trial, participants were presented with a random dot kinematogram (RDK) displayed for 200 ms, with an interval of 2–3 s between RDKs of successive trials. Participants were asked to judge the direction of the dominant dot motion, leftward or rightward. They were given 2 s to press one of two response buttons (up or down) to indicate their decision. The mapping between stimulus (left or right) and response (up or down) varied randomly between blocks, ensuring that, across the whole experiment, there were equal numbers of left-up/right-down and left-down/right-up trials for each difficulty level in each condition.

Trials were run in blocks of six. At the beginning of each block, the response mappings were displayed on the screen during a 2 s instruction period preceding the first trial, and remained on the screen throughout the entire block. The response mappings were indicated by two arrows above and below each other within the dot field aperture, with one arrow pointing right and the other pointing left. In the blocked version, information about the difficulty level of the following block was also shown on the screen (e.g., ‘Difficulty Level is 1’) during the instruction period; while in the mixed version, participants were shown the instructions ‘Prepare for Next Block’. The difficulty level of the six trials in a block remained the same in the blocked design, covering all six difficulty levels every six blocks; while in the mixed design each block contained one trial of each difficulty level randomized within the block.

Participants were given two practice blocks of each condition (coherence and irrelevant-dots) before entering the scanner. Within the scanner, each condition constituted a separate run, each lasting ∼18 min. The order of the two conditions was counterbalanced between participants. In each run there were 60 blocks in total, thus 60 trials per difficulty level, and 30 trials of each left/right motion direction in each difficulty level.

Fig. 2C illustrates coherence and irrelevant-dots conditions across the six difficulty levels. In each RDK there were 64 red dots (RGB channels [112.5, 0, 0]) moving dominantly either left or right for 200 ms (circular aperture with diameter of visual angle ∼8.5∘, dot size = 12 pixels diameter, dot speed = 5 pixels/s, dot lifetime = 5 frames). In the coherence condition, only red dots were present, and difficulty was manipulated by decreasing the percentage of the dots that were moving coherently. The six difficulty levels corresponded to 85%, 60%, 40%, 25%, 15%, or 10% of the dots moving either left or right, while the remaining dots moved in random directions. In the irrelevant-dots condition, the red dots were fixed at 85% coherence, but an additional distractor dot field was present. The distractor dot field consisted of 576 yellow dots, all of which moved randomly, with a net direction of zero (dot size = 12 pixels, dot speed = 7 pixels/s, dot lifetime = 5 frames). Participants were asked to ignore the yellow dots, and judge the dominant motion direction of the red dots. Six levels of difficulty were created by increasing the salience of the yellow distractors (RGB channels [21.25, 21.25, 0], [42.5, 42.5, 0], [85, 85, 0], [127.5, 127.5, 0], [191.25, 191.25, 0], and [255, 255, 0]). These values were selected from previous pilot testing.

Visual stimuli were displayed on a 1920 × 1080 (visual angle 25.16 × 14.31∘) screen with a refresh rate of 60 Hz, which the participants viewed through a mirror placed on top of the head coil. The distance between the participant and screen was approximately 1565 mm. Stimulus presentation was controlled using the Psychophysics Toolbox (Brainard and Vision, 1997) in Matlab (2014a, Mathworks, Natick, WA).

#### Imaging parameters and data analyses

Scanning took place in a 3T Siemens Tim Trio scanner. Functional images were acquired using a multi-band gradient-echo echo-planar imaging (EPI) pulse sequence (TR = 1100 ms, TE = 30 ms, flip angle = 62°, 78 × 78 matrices, slice thickness = 2.5 mm, no gap, voxel size 2.5 mm × 2.5 mm × 2.5 mm, 48 axial slices covering the entire brain, 3 slices acquired at once). The first 10 vol served as dummy scans and were discarded to avoid T1 equilibrium effects. Field maps were collected at the end of the experiment (TR = 400 ms, TE = 5.19 ms/7.65 ms, flip angle = 60°, 64 × 64 matrices, slice thickness = 3 mm, 25% gap, resolution 3 mm isotropic, 32 axial slices). High-resolution anatomical T1-weighted images were acquired for each participant using a 3D MPRAGE sequence (192 axial slices, TR = 2250 ms, TI = 900 ms, TE = 2.99 ms, flip angle = 9°, field of view = 256 mm × 240 mm × 160 mm, matrix dimensions = 256 × 240 × 160, 1 mm isotropic resolution).

The data were preprocessed and analyzed using the automatic analysis (aa) pipelines and modules (Cusack et al., 2014), which called relevant functions from Statistical Parametric Mapping software (SPM 12, http://www.fil.ion.ucl.ac.uk/spm) implemented in Matlab (The MathWorks, Inc., Natick, MA, USA). EPI images were realigned to correct for head motion using rigid-body transformation, unwarped based on the field maps to correct for voxel displacement due to magnetic-field inhomogeneity, and slice time corrected. The mean EPI image was coregistered to the T1 image, and the resulting transformation was applied to the individual EPIs. Then the T1 image was coregistered and normalized to the MNI template, and the resulting transformations were applied to all functional volumes. The functional data were high-pass filtered with a cutoff at 1/128 Hz, and spatial smoothing of 10 mm FWHM was applied.

Statistical analyses were performed first at the individual level using general linear modeling (GLM). For correct trials, separate regressors were created for each combination of condition and difficulty level. As errors can be a strong driver of MD activity (Kiehl et al., 2000; Ullsperger and Von cramon, 2004), error trials and no-response trials were removed using separate regressors. A separate regressor was created for the cue period at the start of each block. All regressors were created by convolving the interval between stimulus onset and response with the canonical hemodynamic response function.

Frontoparietal MD ROIs were taken from Fedorenko et al. (2013) (http://imaging.mrc-cbu.cam.ac.uk/imaging/MDsystem). These included: anterior cingulate and pre-supplementary motor area (ACC/pre-SMA), anterior insula (AI), posterior-dorsal prefrontal cortex (pdLFC), intraparietal sulcus (IPS), and three foci along the middle frontal gyrus (anterior, middle, and posterior MFG). Beta estimates for correct trials were averaged within each ROI and analyzed using a 4-way mixed ANOVA, with factors group (blocked vs. mixed), difficulty type (coherence vs. irrelevant-dots), difficulty level (level 1 ∼ level 6), and ROI (7 MD ROIs). We also performed the same ANOVA on the motion-sensitive visual area MT, using the ROI defined in the SPM anatomy toolbox.

To examine whether individuals showed significant linear effects for difficulty level, we performed a GLM on average activity across the entire MD network, using the MarsBaR toolbox (Brett et al., 2002). Positive linear contrasts (activity increasing with task difficulty) were defined as [-5 -3 -1 +1+3 +5], across difficulty levels 1 to 6, and negative linear contrasts as [+5+3 +1 -1 -3 -5].

An additional whole-brain voxelwise analysis was also performed, to detect any regions that showed a significant positive or negative linear trend for difficulty level, separately for each difficulty type. Activation maps were thresholded at p < 0.05 (FDR corrected).

### Experiment 2

A separate set of participants (n = 24, 18 female, ages 19–30, mean = 24.4) was recruited to perform the coherence and irrelevant-dots conditions along with an additional rule condition. 5 additional participants were excluded (1 because the top of the brain was not acquired, 2 who had head movements > 5 mm, and 2 who had outlier reaction times more than three median absolute deviations above the median). All participants performed the blocked design.

**Fig. 3:**
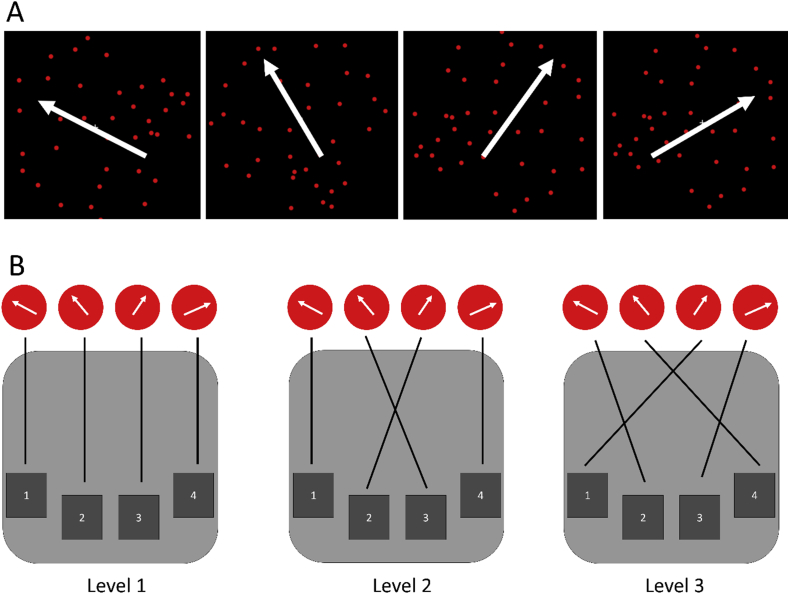
(A) Stimuli in Experiment 2. Red dots moved in one of four possible directions (15°, 65°, 115°, 165°). Additional yellow dots (not shown) were added only in the irrelevant-dots condition. (B) Participants were asked to use a response pad to indicate the direction of the moving dots by pressing the corresponding button. A direct mapping (level 1) was used for coherence and irrelevant-dots conditions, while all 3 rules were used in the rule difficulty condition.

The coherence and irrelevant-dots conditions used a subset (levels 1, 3, and 5) of the same stimuli as previously described, except this time (Fig. 3A) the dots went in one of four directions (15°, 65°, 115°, 165°). The stimuli and response-mappings for the rule condition are illustrated in Fig. 3. Participants responded using the index and middle finger of each hand on the four buttons of a response pad (left hand middle finger for the first button, left hand index finger for the second button, right hand index finger for the third button, and right hand middle finger for the fourth button), with a direct spatial mapping between stimulus direction and response key (Fig. 3B, level 1). The dot fields in the rule difficulty condition had high coherence (85%) and did not include yellow dots. However, rule complexity was manipulated using three different mappings between stimulus direction and response key (Fig. 3B).

At the beginning of each block, participants were presented with a cue indicating the difficulty level of the block. After processing the cue, they were able to press a button to begin a consecutive 6 trials of that condition. Each RDK was presented for 200 ms. Participants had up to 10 s to respond, and after a button was pressed, a 2 s ISI preceded the next trial. At the cue for the next block, participants were given feedback of their performance accuracy as well as mean reaction time for the previous block.

Participants were given two practice blocks of each condition (coherence, irrelevant-dots, and rule) before entering the scanner. Within the scanner, each condition constituted a separate run. The order of the three conditions was counterbalanced across participants. In each run there were 24 blocks in total, thus 48 trials per difficulty level.

## Results

### Experiment 1

#### Behavioral results

**Fig. 4:**
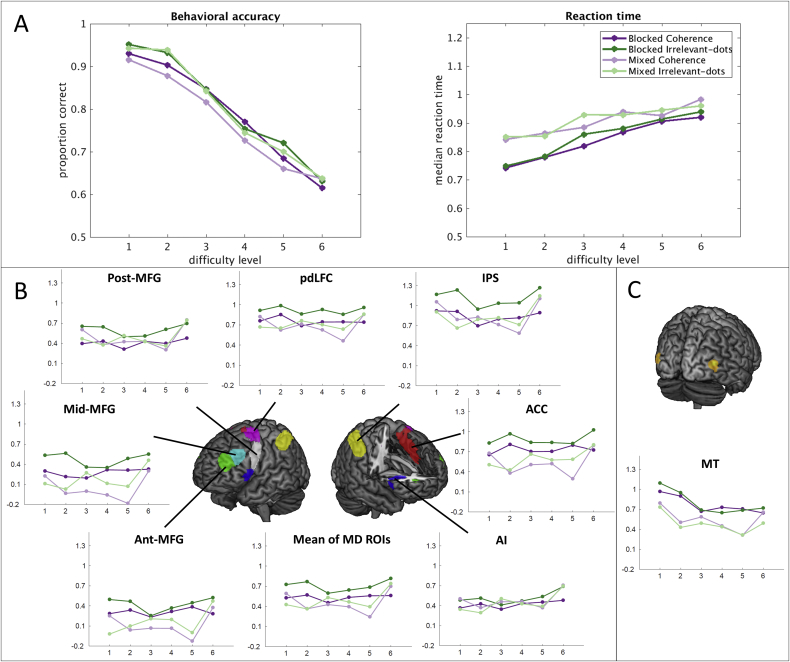
Experiment 1. (A). Behavioral results for each group and condition. Left: accuracy; right: reaction time). (B). ROI results of MD regions. (C). ROI results of MT. Graphs plot the beta values for each condition averaged across bilateral ROIs. Error bars represent the standard error of the mean.

As shown in Fig. 4A, accuracy decreased while reaction times increased with difficulty in both groups. Overall, proportion correct decreased from a mean of 93.5%, to a mean of 63.0% from the easiest to the hardest difficulty level.

3-way ANOVAs of group (blocked vs. mixed) × condition (coherence vs. irrelevant-dots) × difficulty level (level 1 ∼ level 6) were performed on proportion correct and reaction time. The Greenhouse-Geisser correction was used to correct for non-sphericity. For accuracy, there were significant main effects of condition, F(1,38) = 4.1, p = 0.049, with slightly higher accuracy for irrelevant-dots, and difficulty level, F(5,190) = 240.36, p < 0.001. There was no main effect of group, F(1,38) = 0.3, p = 0.61, and no interactions. Analysis of reaction times showed a significant main effect of difficulty level, F(5,190) = 69.6, p < 0.001. The reaction time analysis also showed a significant but small interaction of condition × difficulty level, F(5,190) = 3.8, p < 0.01, but no other effects.

#### fMRI results

Results averaged over bilateral MD regions are shown in Fig. 4B, separately for each condition and group. As a first step, we used 2-way ANOVAs (group × difficulty level) to examine the effect of difficulty separately in each condition, averaged across all MD ROIs. In the coherence condition, the effect of difficulty was not close to significance, F(5,190) = 1.7, p = 0.14. For the irrelevant-dots condition, in contrast, increased activity across difficulty levels was significant, F(5,190) = 3.1, p = 0.01. There were no significant interactions with group.

Next we tested linear increases with difficulty level in each condition separately. For the coherence condition, there were no linear effects of difficulty in the blocked group, F(1,20) = 0.1, p = 0.73, or in the mixed group, F(1,18) = 0.04, p = 0.84. For the irrelevant-dots condition, there was a significant linear trend in the mixed group, F(1,18) = 5.6, p = 0.03, but no effects in the blocked group, F(1,20) = 0.3, p = 0.61.

Next, to compare across groups, conditions and ROIs, we used 4-way ANOVA with factors group (blocked vs. mixed), condition (coherence vs. irrelevant-dots), difficulty level (level 1–level 6), and ROI (7 MD ROIs, averaged across the two hemispheres). This analysis revealed a significant main effect of difficulty level, F(5,190) = 4.1, p < 0.01. Tests of linear contrasts across difficulty levels showed a significant positive trend, F(1,38) = 4.3, p = 0.04. Critically, there was no significant interaction of condition and difficulty level, F(5,190) = 0.3, p = 0.89. A significant interaction between group and difficulty level, F(5,190) = 2.7, p = 0.03, appeared largely driven by increasing activity between difficulty levels 5 and 6 in the mixed group (Fig. 4B). Given the very small difference between these levels in both stimuli and performance, it would seem incautious to interpret this interaction. Finally, the ANOVA showed a significant main effect of ROI, F(6,228) = 37.3, p < 0.001, along with a significant interaction of ROI and difficulty level, F(30,1140) = 2.6, p = < 0.001. Despite this interaction, difficulty trends appeared largely similar across ROIs (Fig. 4B). Separate tests of increasing linear trend for each ROI showed individually significant effects only in AI, F(1,38) = 12.5, p = 0.001, and ACC, F(1,38) = 7.2, p < .02 (the latter not significant after correction for multiple comparisons).

To check that our ROI analysis did not miss important effects elsewhere in the brain, we also carried out a standard whole-brain analysis, combining data for blocked and mixed groups, and testing for a linear increase across difficulty levels (see Methods). For the coherence condition, no significant voxels were found anywhere in the brain. For the irrelevant-dots condition, beyond the expected large increases in visual cortex, the test showed scattered, small regions close to our MD ROIs, including preSMA/ACC, AI, and regions of lateral frontal cortex. In a test for negative linear trends with increasing difficulty, both coherence and irrelevant-dots conditions revealed activity in the visual and sensorimotor regions.

Lastly, we tested for significant linear increases or decreases in individual participants (see Methods), again combining blocked and mixed groups, and using a whole-MD ROI. With 40 participants and an alpha level of .05, for each test we should expect 2 participants to be judged significant by chance. For the coherence condition, there were 10 significant increases but also 8 significant decreases. For the irrelevant-dots condition, there were 17 significant increases and 3 significant decreases. Overall, these results are broadly similar to those from standard random-effects analysis, but further, they suggest a significant degree of heterogeneity between participants.

ROI results for MT are shown in Fig. 4C. In line with prior results (Rees et al., 2000), MT activity generally declined with increasing difficulty of extracting the motion signal. A 3-way ANOVA with factors group (blocked vs. mixed), condition (coherence vs. irrelevant-dots), and difficulty level (level 1–level 6) showed a significant main effect of difficulty level, F(5,190) = 11.7, p < 0.001. There was also a group by level interaction, F(5,190) = 2.9, p = 0.03, reflecting a stronger difficulty effect in the blocked group. No other significant effects were observed. Tests of within-subjects contrasts on difficulty level showed a significant negative linear trend, F(1,38) = 58.6, p < 0.001.

### Experiment 2

#### Behavioral results

**Fig. 5:**
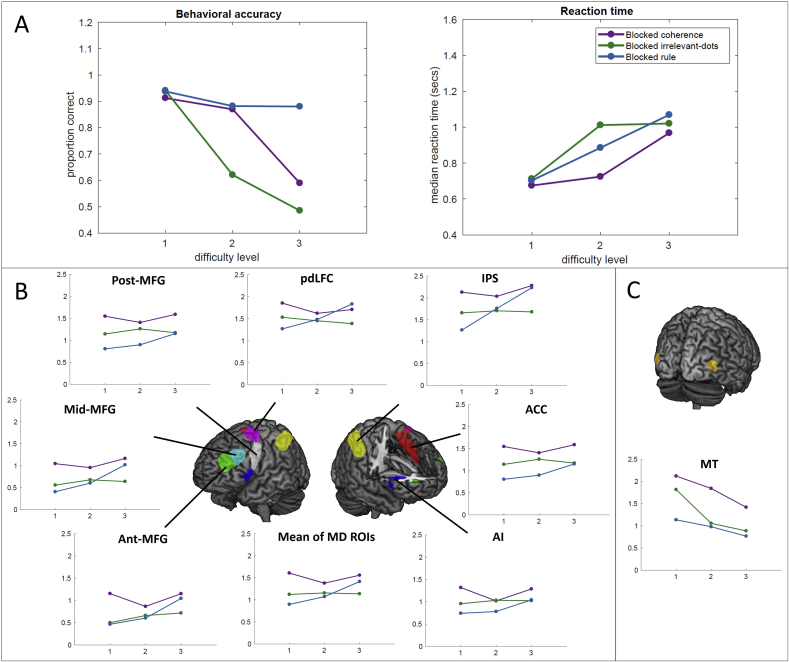
Experiment 2. A. Behavioral results for each group and condition. Left: accuracy; right: reaction time. B. ROI results of MD regions. C. ROI results of MT. Graphs plot the beta values for each condition averaged across bilateral ROIs. Error bars represent the standard error of the mean.

In Experiment 2, participants performed the coherence, irrelevant-dots, and rule conditions. Behavioral data are shown in Fig. 5A. Separate condition (sensory, selection, and rule) × difficulty level (level 1 ∼ level 3) ANOVAs were performed on accuracy and reaction time. For accuracy, there were main effects of difficulty level, F(2,26) = 187.2, p < 0.001, and condition, F(2,46) = 64.2, p < 0.001. There was a significant interaction between condition and difficulty level, F(4,92) = 69.2, p < 0.001, reflecting generally high accuracy across difficulty levels in the rule condition. Reaction times also showed significant main effects of difficulty level, F(2,46) = 112.3, p < 0.001, and condition, F(2,46) = 7.0, p < 0.01, and a significant interaction between condition and difficulty level, F(4,92) = 10.6, p < 0.001.

#### fMRI results

Results for MD regions are shown in Fig. 5B. In this experiment, neither coherence nor irrelevant-dots conditions showed any trend towards increasing activity with increasing difficulty, in contrast to results from the rule condition. In the MD regions, we found significant 2-way interactions of condition and difficulty level, F(4,92) = 2.9, p < .05, and ROI and difficulty level, F(12,276) = 3.6, p < .01, as well as a 3-way interaction of condition, difficulty level, and ROI, F(24,552) = 2.2, p < .05. Further we tested for linear increases across difficulty levels in each condition separately. Difficulty level showed a significant linear trend in the rule condition, F(1,23) = 4.5, p < .05; however, there were no linear trends for either coherence, F(1,23) = 1.1, p = 0.32 or irrelevant -dots F(1,23) = 0.1, p = 0.76.

Whole-brain analysis showed no voxels with a significant linear increase across difficulty levels, either for coherence or irrelevant-dots conditions. For the rule condition, significant effects were found in lateral parietal and lateral frontal cortex bilaterally. In a test for negative linear trends with difficulty, both coherence and irrelevant-dots conditions revealed activity in visual and sensorimotor regions, as well as canonical default mode regions including the medial prefrontal cortex, posterior cingulate cortex, and lateral temporal cortex.

In a whole-MD ROI, tests on individual participants showed 9/24 increases and 6/24 decreases in the coherence condition; the same in the irrelevant-dots condition; and in the rule condition, 14/24 increases and 2/24 decreases.

Results for MT are shown in Fig. 5C. There were significant main effects of difficulty level, F(2,46) = 17.1, p < 0.001, and condition, F(2, 46) = 5.6, p = 0.01, but no interactions.

## Discussion

The characteristic of fronto-parietal “multiple-demand” regions is increased activity for many different kinds of task difficulty. Here, we pursued mixed previous findings suggesting a partial exception – in some cases, MD activity seems largely insensitive to the difficulty of sensory discriminations. To obtain the strongest possible test of such insensitivity, using a motion coherence task, we manipulated two aspects of sensory difficulty over the widest possible range, from very easy to close to chance. We also compared results with difficulty fixed or variable across a block of trials. To ensure good power, across two experiments we tested a total of 64 participants.

Clear results were obtained for a manipulation of motion coherence. Across experiments and mixed or blocked variations of difficulty level, there was no hint of consistently increased MD activity as performance changed from close-to-ceiling to close-to-chance. Following Han and Marois (2013), these results can be interpreted in terms of the distinction drawn by Norman and Bobrow (1975) between data and resource limitations. For motion coherence, the results suggest a scenario similar to that depicted in Fig. 1A: decreasing coherence simply decreases the quality of sensory data, and this cannot be offset by increased attentional focus or top-down control.

An intriguing result was revealed by examining participants individually. Even for motion coherence, there was a clear suggestion of some participants showing a linear increase of MD activity across difficulty levels. These participants were matched, however, by similar numbers showing decreases. It is worth noting that, under the framework of Norman and Bobrow (1975), altered resource allocation is an option whether or not performance is actually resource-limited. When resource allocation has little effect on performance, it may vary idiosyncratically between participants.

Results were less clear for the salience of irrelevant dots. Following Han and Marois (2013), we expected that MD activity might increase with the salience of irrelevant dots, reflecting stronger attempts to focus only on the relevant red dots. Results of Experiment 1 were somewhat in line with this prediction, though even with 40 participants, the effect was not sufficiently strong to differ significantly from the null effect for coherence. In Experiment 2, even irrelevant-dots showed no hint of an overall difficulty effect. Across experiments, results for individual participants again showed a mixture of increases and decreases. Though such variable results lead to no strong interpretation, a reasonable speculation concerns variable strategies. In one extreme case, the participant could make no attempt to separate red and yellow dots, in which case yellow dots simply decrease motion coherence, and results should resemble those of a direct coherence manipulation. Under our presentation conditions, effective top-down separation of the two dot fields may have been hard or impossible to achieve, making unselective processing the dominant strategy. In some cases, however, increasing the salience of yellow dots could have increased top-down attempts to ignore them, reflected in increased MD activity.

Across many different kinds of cognitive demand, it seems that scenarios like that of Fig. 1B are by far the most common. In most cases – including the rule complexity case we used in Experiment 2–increased cognitive demand calls for increased top-down input, ensuring optimal focus on task-relevant processing. The results show, however, that this is not a universal rule. In line with the ideas of Norman and Bobrow (1975), increased attentional focus may be ineffective in overcoming simple limitations of sensory data.

As reviewed in the Introduction, the literature contains mixed findings concerning the MD response to reduced stimulus discriminability. In speeded tasks, for example, strong increases in MD activity have been reported as stimuli to be discriminated are made more similar (Jiang and Kanwisher, 2003; Woolgar et al., 2011). As we have indicated, in general we do not know the shapes of PRFs for different tasks. Rapidly deciding which of four lines is shortest, for example, may have very different attentional requirements from a global judgment of motion direction as used in the present work.

While many studies in the literature show increasing MD activity with increasing task demands, there have also been studies that have showed decreased MD activity (Bor et al., 2003), an inverted U-shape response (Callicott et al., 1999; Linden et al., 2003), or a plateau after a certain difficulty level (Marois and Ivanoff, 2005; Todd and Marois, 2004; Mitchell and Cusack, 2008). For example, Linden et al. (2003) and Callicott et al. (1999) found that the frontal-parietal network initially showed increased activation with increased working memory load, but decreased in the highest load condition close or beyond the limit of capacity. In our data there was no suggestion of an inverted-U profile; if anything, in some conditions there was a decrease in MD activity over the first few difficulty levels (e.g. Experiment 2, coherence condition). Our data suggest no decrease of MD activity as sensory limits make a task impossible to perform well.

One factor affecting MD recruitment might be advance knowledge of difficulty level. The neural basis of expectation in perceptual tasks has been shown to involve top-down modulation of frontal and parietal cortices to enhance sensory processing in the visual cortex (Giesbrecht et al., 2006; Kastner et al., 1999; Kok et al., 2012; Sylvester et al., 2009). Furthermore, Manelis and Reder (2015) recently demonstrated using MVPA that the regions involved in a working memory task are the same regions that contain information about the upcoming task difficulty during task preparation. It is therefore possible in our study that participants in the blocked group could have decided to increase attentional effort in an attempt to compensate for anticipated perceptual difficulty. Our data, however, suggested little notable difference between mixed and blocked conditions.

Activity across the MD network is increased by many different kinds of cognitive demand (Duncan and Owen, 2000; Fedorenko et al., 2013). In contrast to this “multiple demand” pattern, the present results show little or no consistent effect of sensory manipulations in a simple motion coherence task. As suggested by Han and Marois (2013), results may reflect the degree to which performance can be improved by increasing attentional investment. When simple sensory decisions are largely data-limited, decreased accuracy cannot be compensated by increased attention, leading to little or no enhancement of MD activity.
